# (*E*)-1-(4-Benzhydrylpiperazin-1-yl)-3-(2-eth­oxy­phen­yl)prop-2-en-1-one

**DOI:** 10.1107/S1600536811045922

**Published:** 2011-11-05

**Authors:** Yan Zhong, XiaoPing Zhang, Bin Wu

**Affiliations:** a School of Chemistry and Chemical Engineering, Southeast University, Sipailou No. 2 Nanjing, Nanjing 210096, People’s Republic of China; b Centre of Laboratory Animals, Nanjing Medical University, Hanzhong Road No. 140 Nanjing, Nanjing 210029, People’s Republic of China; c School of Pharmacy, Nanjing Medical University, Hanzhong Road No. 140 Nanjing, Nanjing 210029, People’s Republic of China

## Abstract

In the title mol­ecule, C_28_H_30_N_2_O_2_, the piperazine ring adopts a chair conformation and the C=C bond exhibits an *E* conformation. The dihedral angle between the terminal phenyl rings is 71.4 (2). In the crystal, mol­ecules are linked by C—H⋯O hydrogen bonds, forming [010] chains.

## Related literature

For properties of cinnamic acid derivatives, see: Shi *et al.* (2005 ▶); Qian *et al.* (2010 ▶). For the synthesis, see: Wu *et al.* (2008 ▶). For related structures, see: Mouillé *et al.* (1975) ▶; Zhong *et al.* (2011 ▶).
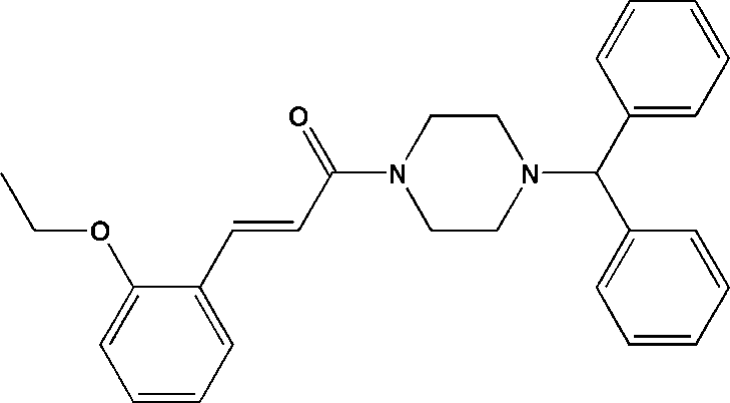


## Experimental

### 

#### Crystal data

C_28_H_30_N_2_O_2_
*M* *_r_* = 426.54
Monoclinic, 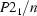

*a* = 11.858 (2) Å
*b* = 12.786 (3) Å
*c* = 16.044 (3) Å
β = 94.63 (3)°
*V* = 2424.6 (8) Å^3^
*Z* = 4
Mo *K*α radiation
μ = 0.07 mm^−1^
*T* = 293 K
0.30 × 0.20 × 0.10 mm

#### Data collection

Enraf–Nonius CAD-4 diffractometer
4673 measured reflections
4446 independent reflections
2022 reflections with *I* > 2σ(*I*)
*R*_int_ = 0.063
3 standard reflections every 200 reflections  intensity decay: 1%

#### Refinement

*R*[*F* ^2^ > 2σ(*F* ^2^)] = 0.077
*wR*(*F* ^2^) = 0.155
*S* = 1.00
4446 reflections
265 parameters
H-atom parameters constrained
Δρ_max_ = 0.13 e Å^−3^
Δρ_min_ = −0.22 e Å^−3^

### 

Data collection: *CAD-4 EXPRESS* (Enraf–Nonius, 1989 ▶); cell refinement: *CAD-4 EXPRESS*; data reduction: *XCAD4* (Harms & Wocadlo, 1995 ▶); program(s) used to solve structure: *SHELXS97* (Sheldrick, 2008 ▶); program(s) used to refine structure: *SHELXL97* (Sheldrick, 2008 ▶); molecular graphics: *SHELXL97* (Sheldrick, 2008 ▶); software used to prepare material for publication: *SHELXL97*.

## Supplementary Material

Crystal structure: contains datablock(s) I, global. DOI: 10.1107/S1600536811045922/hb6487sup1.cif

Structure factors: contains datablock(s) I. DOI: 10.1107/S1600536811045922/hb6487Isup2.hkl

Supplementary material file. DOI: 10.1107/S1600536811045922/hb6487Isup3.cml

Additional supplementary materials:  crystallographic information; 3D view; checkCIF report

## Figures and Tables

**Table 1:** Hydrogen-bond geometry (Å, °)

*D*—H⋯*A*	*D*—H	H⋯*A*	*D*⋯*A*	*D*—H⋯*A*
C11—H11*A*⋯O1^i^	0.93	2.54	3.343 (6)	146

Symmetry code: (i) 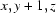.

**Fig. 1.**
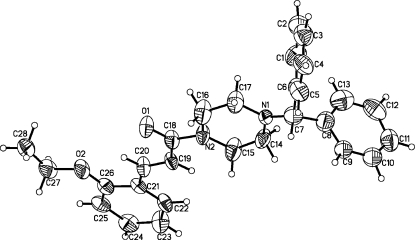
The molecular structure of the title compound with displacement ellipsoids for non-H atoms drawn at the 70% probability level.

**Fig. 2.**
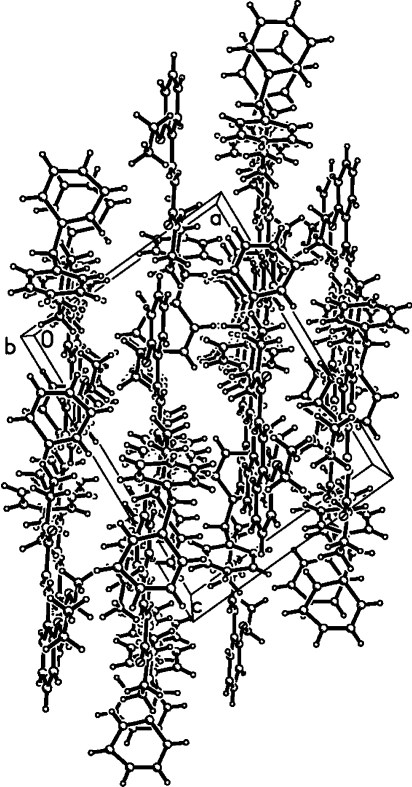
Packing diagram of the title compound.

## supplementary crystallographic information

### Comment

Many compounds containing a cinnamoyl moiety have been reported to possess antimicrobial, anticancer and neuroprotective activities (Shi *et al.*, 2005; Qian *et al.*, 2010). We report herein the crystal structure of the title compound. The molecule of the title compound exists an E configulation with respect to the C19=C20 ethene bond [1.291 (4)] and the torsion angle C18—C19—C20—C21 = 177.2 (3). The piperazine ring adopts a chair conformation. In the crystal, molecules are linked by C—H···O interactions.

### Experimental

The synthesis follows the method of Wu *et al.* (2008). The title compound was prepared by stirring a mixture of (*E*)-3-(2-ethoxyphenyl)acrylic acid (0.769 g; 4 mmol), dimethyl sulfoxide (2 ml) and dichloromethane (30 ml) for 6 h at room temperature. The solvent was removed under reduced pressure. The residue was dissolved in acetone (15 ml) and reacted with 1-benzhydrylpiperazine (1.514 g; 6 mmol) in the presence of triethylamine (5 ml) for 12 h at room temperature. The resultant mixture was cooled. The solid, (*E*)-1-(4-benzhydrylpiperazin-1-yl)-3-(2-ethoxyphenyl)prop-2-en-1-one obtained was filtered and was recrystallized from ethanol. Pale-yellow blocks were grown in ethyl acetate and hexane by a slow evaporation at room temperature.

### Refinement

All non-hydrogen atoms were refined anisotropically. All hydrogen atoms were positioned geometrically with C—H distances ranging from 0.93 Å to 0.98 Å and refined as riding on their parent atoms with *U*ĩso~(H) = 1.2 or 1.5*U*~eq~ of the carrier atom.

### Crystal data

**Table:**

C_28_H_30_N_2_O_2_	*F*(000) = 912
*M**_r_* = 426.54	*D*_x_ = 1.169 Mg m^−^^3^
Monoclinic, *P*2_1_/*n*	Mo *K*α radiation, λ = 0.71073 Å
Hall symbol: -P 2yn	Cell parameters from 25 reflections
*a* = 11.858 (2) Å	θ = 9–12°
*b* = 12.786 (3) Å	µ = 0.07 mm^−^^1^
*c* = 16.044 (3) Å	*T* = 293 K
β = 94.63 (3)°	Block, pale-yellow
*V* = 2424.6 (8) Å^3^	0.30 × 0.20 × 0.10 mm
*Z* = 4	

### Data collection

**Table:**

Enraf–Nonius CAD-4 diffractometer	*R*_int_ = 0.063
Radiation source: fine-focus sealed tube	θ_max_ = 25.4°, θ_min_ = 2.0°
graphite	*h* = 0→14
ω/2θ scans	*k* = 0→15
4673 measured reflections	*l* = −19→19
4446 independent reflections	3 standard reflections every 200 reflections
2022 reflections with *I* > 2σ(*I*)	intensity decay: 1%

### Refinement

**Table:**

Refinement on *F*^2^	Primary atom site location: structure-invariant direct methods
Least-squares matrix: full	Secondary atom site location: difference Fourier map
*R*[*F*^2^ > 2σ(*F*^2^)] = 0.077	Hydrogen site location: inferred from neighbouring sites
*wR*(*F*^2^) = 0.155	H-atom parameters constrained
*S* = 1.00	*w* = 1/[σ^2^(*F*_o_^2^) + (0.030*P*)^2^ + 1.550*P*] where *P* = (*F*_o_^2^ + 2*F*_c_^2^)/3
4446 reflections	(Δ/σ)_max_ < 0.001
265 parameters	Δρ_max_ = 0.13 e Å^−^^3^
0 restraints	Δρ_min_ = −0.22 e Å^−^^3^

### Special details

**Table:**

Geometry. All e.s.d.'s (except the e.s.d. in the dihedral angle between two l.s. planes) are estimated using the full covariance matrix. The cell e.s.d.'s are taken into account individually in the estimation of e.s.d.'s in distances, angles and torsion angles; correlations between e.s.d.'s in cell parameters are only used when they are defined by crystal symmetry. An approximate (isotropic) treatment of cell e.s.d.'s is used for estimating e.s.d.'s involving l.s. planes.
Refinement. Refinement of *F*^2^ against ALL reflections. The weighted *R*-factor *wR* and goodness of fit *S* are based on *F*^2^, conventional *R*-factors *R* are based on *F*, with *F* set to zero for negative *F*^2^. The threshold expression of *F*^2^ > σ(*F*^2^) is used only for calculating *R*-factors(gt) *etc*. and is not relevant to the choice of reflections for refinement. *R*-factors based on *F*^2^ are statistically about twice as large as those based on *F*, and *R*- factors based on ALL data will be even larger.

### Fractional atomic coordinates and isotropic or equivalent isotropic displacement parameters (Å^2^)

**Table:**

	*x*	*y*	*z*	*U*_iso_*/*U*_eq_	
O1	0.2436 (2)	0.32431 (17)	0.49193 (13)	0.0823 (8)	
N1	0.2112 (2)	0.69070 (19)	0.57757 (17)	0.0641 (7)	
C1	0.0062 (5)	0.7832 (3)	0.6573 (3)	0.1159 (15)	
H1A	−0.0014	0.7739	0.5996	0.139*	
C2	−0.0941 (4)	0.7913 (3)	0.6932 (3)	0.103	
H2A	−0.1671	0.7873	0.6674	0.124*	
C3	−0.0616 (5)	0.8068 (3)	0.7764 (3)	0.1158 (15)	
H3A	−0.1233	0.8218	0.8067	0.139*	
C4	0.0365 (5)	0.8061 (4)	0.8263 (3)	0.1107 (15)	
H4A	0.0396	0.8135	0.8841	0.133*	
C5	0.1251 (5)	0.7940 (3)	0.7854 (4)	0.1213 (16)	
H5A	0.1962	0.7901	0.8142	0.146*	
C6	0.1140 (4)	0.7863 (3)	0.6906 (4)	0.1098 (17)	
O2	0.3846 (2)	0.12578 (18)	0.28272 (15)	0.0852 (8)	
N2	0.2698 (3)	0.4974 (2)	0.50411 (17)	0.0790 (9)	
C7	0.2064 (3)	0.7757 (3)	0.6338 (3)	0.095	
H7A	0.2752	0.7684	0.6715	0.114*	
C8	0.2259 (4)	0.8738 (3)	0.5848 (3)	0.1061 (14)	
C9	0.3187 (3)	0.9292 (3)	0.6198 (2)	0.084	
H9A	0.3618	0.9034	0.6663	0.100*	
C10	0.3444 (4)	1.0185 (3)	0.5861 (3)	0.097	
H10A	0.4150	1.0458	0.6040	0.117*	
C11	0.2883 (5)	1.0710 (4)	0.5344 (3)	0.1141 (15)	
H11A	0.3101	1.1387	0.5221	0.137*	
C12	0.1862 (5)	1.0252 (5)	0.4921 (3)	0.1197 (17)	
H12A	0.1442	1.0597	0.4490	0.144*	
C13	0.1550 (4)	0.9251 (5)	0.5206 (3)	0.1267 (17)	
H13A	0.0891	0.8930	0.4979	0.152*	
C14	0.2828 (3)	0.6824 (3)	0.5164 (3)	0.1075 (15)	
H14A	0.3513	0.7181	0.5377	0.129*	
H14B	0.2498	0.7249	0.4707	0.129*	
C15	0.3189 (4)	0.5913 (3)	0.4794 (3)	0.1085 (15)	
H15A	0.4002	0.5859	0.4912	0.130*	
H15B	0.3041	0.5981	0.4193	0.130*	
C16	0.1949 (4)	0.4975 (3)	0.5695 (3)	0.1160 (16)	
H16A	0.1277	0.4580	0.5508	0.139*	
H16B	0.2315	0.4614	0.6175	0.139*	
C17	0.1613 (4)	0.5974 (3)	0.5953 (3)	0.1131 (16)	
H17A	0.0823	0.6043	0.5749	0.136*	
H17B	0.1627	0.5941	0.6557	0.136*	
C18	0.2861 (3)	0.4023 (3)	0.4683 (2)	0.0757 (11)	
C19	0.3575 (3)	0.4008 (2)	0.39672 (19)	0.0670 (9)	
H19A	0.3894	0.4628	0.3797	0.080*	
C20	0.3765 (3)	0.3152 (2)	0.35741 (18)	0.0720 (10)	
H20A	0.3453	0.2550	0.3786	0.086*	
C21	0.4405 (3)	0.3001 (3)	0.28366 (19)	0.0637 (9)	
C22	0.4967 (3)	0.3810 (3)	0.2482 (2)	0.0733 (10)	
H22A	0.4923	0.4476	0.2711	0.088*	
C23	0.5596 (3)	0.3673 (3)	0.1799 (2)	0.0918 (13)	
H23A	0.5981	0.4232	0.1584	0.110*	
C24	0.5641 (3)	0.2701 (3)	0.1446 (2)	0.0873 (12)	
H24A	0.6081	0.2590	0.1000	0.105*	
C25	0.5004 (3)	0.1846 (3)	0.1769 (2)	0.0829 (11)	
H25A	0.4989	0.1199	0.1504	0.100*	
C26	0.4417 (3)	0.1990 (3)	0.2469 (2)	0.0721 (10)	
C27	0.3835 (3)	0.0218 (3)	0.2493 (2)	0.0919 (12)	
H27A	0.4600	−0.0052	0.2505	0.110*	
H27B	0.3516	0.0223	0.1917	0.110*	
C28	0.3142 (3)	−0.0455 (3)	0.3005 (3)	0.1021 (13)	
H28A	0.3140	−0.1158	0.2794	0.153*	
H28B	0.2381	−0.0194	0.2977	0.153*	
H28C	0.3457	−0.0449	0.3575	0.153*	

### Atomic displacement parameters (Å^2^)

**Table:**

	*U*^11^	*U*^22^	*U*^33^	*U*^12^	*U*^13^	*U*^23^
O1	0.129 (2)	0.0544 (14)	0.0689 (15)	−0.0102 (14)	0.0430 (14)	−0.0047 (12)
N1	0.0595 (18)	0.0530 (16)	0.0809 (19)	0.0014 (14)	0.0131 (15)	−0.0082 (15)
C1	0.119 (4)	0.121 (4)	0.111 (4)	−0.025 (3)	0.028 (3)	0.009 (3)
C2	0.103	0.103	0.103	0.000	0.008	0.000
C3	0.128 (4)	0.102 (3)	0.120 (4)	0.010 (3)	0.024 (3)	−0.005 (3)
C4	0.114 (4)	0.110 (4)	0.110 (4)	0.029 (3)	0.024 (3)	−0.014 (3)
C5	0.112 (4)	0.097 (3)	0.153 (5)	0.016 (3)	0.003 (4)	−0.006 (3)
C6	0.102 (4)	0.077 (3)	0.162 (5)	0.004 (3)	0.080 (4)	−0.032 (3)
O2	0.104 (2)	0.0662 (15)	0.0907 (18)	0.0147 (14)	0.0390 (15)	−0.0212 (14)
N2	0.113 (2)	0.0495 (16)	0.081 (2)	−0.0052 (16)	0.0478 (18)	−0.0007 (15)
C7	0.095	0.095	0.095	0.000	0.008	0.000
C8	0.107 (4)	0.090 (3)	0.125 (4)	−0.007 (3)	0.032 (3)	−0.010 (3)
C9	0.084	0.084	0.084	0.000	0.007	0.000
C10	0.097	0.097	0.097	0.000	0.008	0.000
C11	0.137 (4)	0.088 (3)	0.120 (4)	0.009 (3)	0.029 (3)	0.013 (3)
C12	0.124 (5)	0.146 (5)	0.090 (3)	0.046 (4)	0.011 (3)	−0.005 (4)
C13	0.113 (4)	0.154 (5)	0.114 (4)	−0.005 (4)	0.019 (3)	−0.032 (4)
C14	0.104 (3)	0.066 (2)	0.162 (4)	−0.023 (2)	0.068 (3)	−0.001 (3)
C15	0.153 (4)	0.074 (3)	0.108 (3)	−0.016 (3)	0.067 (3)	−0.019 (3)
C16	0.171 (4)	0.079 (3)	0.110 (3)	−0.009 (3)	0.085 (3)	−0.013 (2)
C17	0.136 (4)	0.072 (3)	0.143 (4)	−0.006 (3)	0.088 (3)	−0.010 (3)
C18	0.098 (3)	0.059 (2)	0.076 (2)	0.006 (2)	0.047 (2)	−0.0040 (19)
C19	0.084 (2)	0.0472 (18)	0.075 (2)	0.0158 (17)	0.0369 (19)	0.0063 (17)
C20	0.114 (3)	0.0499 (19)	0.057 (2)	0.0050 (19)	0.039 (2)	0.0019 (16)
C21	0.064 (2)	0.068 (2)	0.061 (2)	0.0222 (18)	0.0102 (17)	0.0044 (18)
C22	0.094 (3)	0.063 (2)	0.066 (2)	0.023 (2)	0.024 (2)	0.0038 (18)
C23	0.137 (4)	0.080 (3)	0.065 (2)	0.000 (3)	0.046 (2)	0.007 (2)
C24	0.090 (3)	0.112 (3)	0.065 (2)	0.012 (3)	0.034 (2)	0.005 (2)
C25	0.070 (2)	0.101 (3)	0.082 (3)	0.024 (2)	0.030 (2)	−0.011 (2)
C26	0.074 (3)	0.075 (2)	0.069 (2)	0.016 (2)	0.013 (2)	0.003 (2)
C27	0.097 (3)	0.093 (3)	0.087 (3)	0.018 (2)	0.014 (2)	−0.037 (2)
C28	0.097 (3)	0.083 (3)	0.129 (4)	0.014 (2)	0.027 (3)	−0.017 (3)

### Geometric parameters (Å, °)

**Table:**

O1—C18	1.194 (4)	C13—H13A	0.9300
N1—C14	1.352 (4)	C14—C15	1.391 (4)
N1—C17	1.372 (4)	C14—H14A	0.9700
N1—C7	1.416 (4)	C14—H14B	0.9700
C1—C6	1.347 (6)	C15—H15A	0.9700
C1—C2	1.367 (6)	C15—H15B	0.9700
C1—H1A	0.9300	C16—C17	1.410 (5)
C2—C3	1.373 (6)	C16—H16A	0.9700
C2—H2A	0.9300	C16—H16B	0.9700
C3—C4	1.357 (6)	C17—H17A	0.9700
C3—H3A	0.9300	C17—H17B	0.9700
C4—C5	1.292 (6)	C18—C19	1.480 (4)
C4—H4A	0.9300	C19—C20	1.291 (4)
C5—C6	1.519 (6)	C19—H19A	0.9300
C5—H5A	0.9300	C20—C21	1.470 (4)
C6—C7	1.487 (5)	C20—H20A	0.9300
O2—C26	1.315 (4)	C21—C22	1.378 (4)
O2—C27	1.434 (4)	C21—C26	1.421 (4)
N2—C18	1.366 (4)	C22—C23	1.385 (4)
N2—C15	1.405 (4)	C22—H22A	0.9300
N2—C16	1.428 (4)	C23—C24	1.368 (5)
C7—C8	1.508 (6)	C23—H23A	0.9300
C7—H7A	0.9800	C24—C25	1.449 (5)
C8—C9	1.389 (5)	C24—H24A	0.9300
C8—C13	1.435 (6)	C25—C26	1.380 (4)
C9—C10	1.310 (5)	C25—H25A	0.9300
C9—H9A	0.9300	C27—C28	1.482 (5)
C10—C11	1.222 (5)	C27—H27A	0.9700
C10—H10A	0.9300	C27—H27B	0.9700
C11—C12	1.461 (6)	C28—H28A	0.9600
C11—H11A	0.9300	C28—H28B	0.9600
C12—C13	1.419 (6)	C28—H28C	0.9600
C12—H12A	0.9300		
			
C14—N1—C17	112.8 (3)	C14—C15—H15A	108.2
C14—N1—C7	125.6 (3)	N2—C15—H15A	108.2
C17—N1—C7	119.7 (3)	C14—C15—H15B	108.2
C6—C1—C2	131.4 (5)	N2—C15—H15B	108.2
C6—C1—H1A	114.3	H15A—C15—H15B	107.3
C2—C1—H1A	114.3	C17—C16—N2	115.1 (3)
C1—C2—C3	103.6 (4)	C17—C16—H16A	108.5
C1—C2—H2A	128.2	N2—C16—H16A	108.5
C3—C2—H2A	128.2	C17—C16—H16B	108.5
C4—C3—C2	137.0 (5)	N2—C16—H16B	108.5
C4—C3—H3A	111.5	H16A—C16—H16B	107.5
C2—C3—H3A	111.5	N1—C17—C16	126.2 (3)
C5—C4—C3	113.3 (5)	N1—C17—H17A	105.8
C5—C4—H4A	123.4	C16—C17—H17A	105.8
C3—C4—H4A	123.4	N1—C17—H17B	105.8
C4—C5—C6	120.5 (5)	C16—C17—H17B	105.8
C4—C5—H5A	119.7	H17A—C17—H17B	106.2
C6—C5—H5A	119.7	O1—C18—N2	122.2 (3)
C1—C6—C7	118.5 (5)	O1—C18—C19	121.2 (3)
C1—C6—C5	113.7 (4)	N2—C18—C19	116.6 (3)
C7—C6—C5	127.7 (5)	C20—C19—C18	121.6 (3)
C26—O2—C27	119.1 (3)	C20—C19—H19A	119.2
C18—N2—C15	124.4 (3)	C18—C19—H19A	119.2
C18—N2—C16	115.3 (3)	C19—C20—C21	128.8 (3)
C15—N2—C16	120.2 (3)	C19—C20—H20A	115.6
N1—C7—C6	122.0 (4)	C21—C20—H20A	115.6
N1—C7—C8	106.9 (3)	C22—C21—C26	119.4 (3)
C6—C7—C8	113.3 (4)	C22—C21—C20	122.1 (3)
N1—C7—H7A	104.3	C26—C21—C20	118.5 (3)
C6—C7—H7A	104.3	C21—C22—C23	122.7 (3)
C8—C7—H7A	104.3	C21—C22—H22A	118.7
C9—C8—C13	117.2 (4)	C23—C22—H22A	118.7
C9—C8—C7	111.3 (5)	C24—C23—C22	119.0 (3)
C13—C8—C7	130.4 (5)	C24—C23—H23A	120.5
C10—C9—C8	118.7 (4)	C22—C23—H23A	120.5
C10—C9—H9A	120.6	C23—C24—C25	119.9 (3)
C8—C9—H9A	120.6	C23—C24—H24A	120.1
C11—C10—C9	128.8 (5)	C25—C24—H24A	120.1
C11—C10—H10A	115.6	C26—C25—C24	120.1 (3)
C9—C10—H10A	115.6	C26—C25—H25A	119.9
C10—C11—C12	118.8 (5)	C24—C25—H25A	119.9
C10—C11—H11A	120.6	O2—C26—C25	124.9 (3)
C12—C11—H11A	120.6	O2—C26—C21	116.3 (3)
C13—C12—C11	116.0 (5)	C25—C26—C21	118.8 (3)
C13—C12—H12A	122.0	O2—C27—C28	108.6 (3)
C11—C12—H12A	122.0	O2—C27—H27A	110.0
C12—C13—C8	119.4 (5)	C28—C27—H27A	110.0
C12—C13—H13A	120.3	O2—C27—H27B	110.0
C8—C13—H13A	120.3	C28—C27—H27B	110.0
N1—C14—C15	127.5 (3)	H27A—C27—H27B	108.3
N1—C14—H14A	105.4	C27—C28—H28A	109.5
C15—C14—H14A	105.4	C27—C28—H28B	109.5
N1—C14—H14B	105.4	H28A—C28—H28B	109.5
C15—C14—H14B	105.4	C27—C28—H28C	109.5
H14A—C14—H14B	106.0	H28A—C28—H28C	109.5
C14—C15—N2	116.4 (3)	H28B—C28—H28C	109.5
			
C6—C1—C2—C3	1.4 (7)	C18—N2—C15—C14	172.9 (4)
C1—C2—C3—C4	−7.1 (8)	C16—N2—C15—C14	−5.5 (6)
C2—C3—C4—C5	5.4 (9)	C18—N2—C16—C17	−168.4 (4)
C3—C4—C5—C6	2.2 (7)	C15—N2—C16—C17	10.1 (6)
C2—C1—C6—C7	−179.6 (4)	C14—N1—C17—C16	13.8 (7)
C2—C1—C6—C5	4.0 (8)	C7—N1—C17—C16	−151.5 (5)
C4—C5—C6—C1	−5.9 (7)	N2—C16—C17—N1	−15.2 (8)
C4—C5—C6—C7	178.0 (4)	C15—N2—C18—O1	179.2 (4)
C14—N1—C7—C6	170.6 (4)	C16—N2—C18—O1	−2.4 (6)
C17—N1—C7—C6	−26.1 (6)	C15—N2—C18—C19	−2.2 (6)
C14—N1—C7—C8	38.0 (5)	C16—N2—C18—C19	176.2 (3)
C17—N1—C7—C8	−158.7 (4)	O1—C18—C19—C20	−0.1 (6)
C1—C6—C7—N1	−53.6 (6)	N2—C18—C19—C20	−178.7 (4)
C5—C6—C7—N1	122.3 (5)	C18—C19—C20—C21	177.2 (3)
C1—C6—C7—C8	76.4 (5)	C19—C20—C21—C22	3.7 (6)
C5—C6—C7—C8	−107.7 (5)	C19—C20—C21—C26	−174.7 (4)
N1—C7—C8—C9	−122.0 (4)	C26—C21—C22—C23	−3.2 (5)
C6—C7—C8—C9	100.8 (5)	C20—C21—C22—C23	178.5 (3)
N1—C7—C8—C13	70.4 (6)	C21—C22—C23—C24	1.7 (6)
C6—C7—C8—C13	−66.7 (6)	C22—C23—C24—C25	2.3 (6)
C13—C8—C9—C10	−8.2 (6)	C23—C24—C25—C26	−4.7 (6)
C7—C8—C9—C10	−177.6 (3)	C27—O2—C26—C25	1.5 (5)
C8—C9—C10—C11	12.5 (7)	C27—O2—C26—C21	−179.3 (3)
C9—C10—C11—C12	−10.9 (7)	C24—C25—C26—O2	−177.6 (3)
C10—C11—C12—C13	5.5 (7)	C24—C25—C26—C21	3.2 (5)
C11—C12—C13—C8	−2.8 (6)	C22—C21—C26—O2	−178.7 (3)
C9—C8—C13—C12	4.3 (6)	C20—C21—C26—O2	−0.2 (5)
C7—C8—C13—C12	171.3 (4)	C22—C21—C26—C25	0.6 (5)
C17—N1—C14—C15	−8.2 (7)	C20—C21—C26—C25	179.1 (3)
C7—N1—C14—C15	156.1 (5)	C26—O2—C27—C28	179.8 (3)
N1—C14—C15—N2	4.7 (8)		

### Hydrogen-bond geometry (Å, °)

**Table:**

*D*—H···*A*	*D*—H	H···*A*	*D*···*A*	*D*—H···*A*
C11—H11A···O1^i^	0.93	2.54	3.343 (6)	146

Symmetry codes: (i) *x*, *y*+1, *z*.
